# USE OF OFF-LABEL MEDICATIONS IN A NEONATAL INTENSIVE CARE UNIT

**DOI:** 10.1590/1984-0462/2021/39/2020063

**Published:** 2021-01-11

**Authors:** Erica Inez Alves Koszma, Ana Jovina Barreto Bispo, Isabelle Araujo de Oliveira Santana, Catharine Natielle Oliveira Dias Belarmino dos Santos

**Affiliations:** a Universidade Tiradentes, Aracaju, SE, Brazil.

**Keywords:** Infant, newborn, Drug utilization, Off-label use, Intensive Care Units, Neonatal, Recém-nascidos, Uso de medicamentos, Uso off-label, Unidade de Terapia Intensiva Neonatal

## Abstract

**Objective:**

This paper aims to analyze the use of off label (OL) medicines, according to the National Regulatory Agency, in a neonatal intensive care unit of a high-risk maternity hospital in Northeast Brazil.

**Methods:**

A cross-sectional study was carried out, using a convenience sample of newborns that used mechanical ventilation at the Intensive Care Unit. As a reference, OL medications were considered for those without an approval for newborn usage by the Brazilian Health Regulatory Agency (*Agência Nacional de Vigilância Sanitária* - ANVISA) and by the Food and Drugs Administration (FDA).

**Results:**

The sample consisted of 158 newborns, 58.3% male, 87.7% premature, and 70.2% of low or very low birth weight. According to ANVISA, 440 out of the 1,167 prescriptions analyzed were OL, with 98.1% of newborns exposed to at least one of these drugs. According to the FDA, 484 prescriptions were OL, with 75.8% of newborns exposed to at least one of them. Anti-infectives were the most prescribed OL medications. Neonates who presented respiratory failure and pneumonia used these drugs more often; and there was no relation between their use and the number of deaths.

**Conclusions:**

Nearly all newborns at the Intensive Care Units, mainly preterm infants, are exposed to at least one off-label (OL) medication during hospital stay, according to the national and international regulatory agencies. No association was found between off-label prescriptions and the frequency of complications or neonatal deaths.

## RESUMO

**Objetivo:**

Analisar o uso de medicamentos *off-label* (OL), segundo a agência reguladora nacional, em unidade de terapia intensiva neonatal de uma maternidade de alto risco em Aracaju.

**Métodos:**

Foi realizado um estudo transversal, utilizando amostra de conveniência de recém-nascidos (RN) da Unidade Intensiva, que fizeram uso de ventilação mecânica. Consideramos OL o medicamento que não era liberado para uso em RN nos bulários eletrônicos da Agência Nacional de Vigilância Sanitária (ANVISA) e da U.S. Food and Drug Administration (FDA).

**Resultados:**

A amostra consistiu de 158 neonatos, sendo 58,3% do sexo masculino, 87,7% prematuros e 70,2% com baixo ou muito baixo peso. De acordo com a ANVISA, das 1.167 prescrições analisadas, 440 foram OL, com 98,1% dos RN expostos a pelo menos um desses medicamentos*.* Já para a FDA, 484 prescrições foram OL, com 75,8% dos neonatos expostos a pelo menos um deles. As medicações OL mais prescritas foram os anti-infecciosos. Neonatos que apresentaram insuficiência respiratória e pneumonia fizeram mais uso deles e não houve relação entre o seu uso e o número de óbitos.

**Conclusões:**

Quase todos os RN internados, principalmente os prematuros, foram expostos a pelo menos um medicamento OL, de acordo com a agência reguladora nacional e internacional, durante a internação. Entretanto, isso não teve relação com a frequência de complicações nem de óbitos neonatais.

## INTRODUCTION

According to the World Health Organization (WHO), unsafe practices and medication errors are two of the main causes of preventable damage to health systems worldwide.^1^ Half of the drugs used worldwide are estimated to be prescribed in an inadequate way, with consequences that include increased morbidity and mortality, health expenditures and wasted resources.^2^ Therefore, the use of dubious and inconsistent medications is recognized as a major global problem.

Therapy with off-label (OL) medications is included in this scenario and occurs when a drug is prescribed under conditions different from those recommended in the drug prescription leaflet, regarding therapeutic indication, route and frequency of administration, dosage, patient’s age, and presentation.^3^ Despite this, the use of OL can be considered legal unless it violates ethical guidelines or national security regulations,^4^ and is often performed by doctors focused on the individual benefit of patients, especially in populations that are not well represented in clinical trials, such as children and pregnant women.^5^

In this context, the Neonatal Intensive Care Unit (NICU) is the place that concentrates the main human and material resources needed to provide uninterrupted support to newborns (NBs) who require intensive care.^6^ Such care almost always involves the use of several medications, many of them without the proper drug description leaflet for use in the first month of life. In 2017, the Brazilian Ministry of Health published a document on pharmaceutical assistance in Pediatrics, which showed the little participation of children in research, with only 8% of the total number of studies related to medicines, both in the global scenario and in Brazil.^7^ Given this reality, the insufficiency of scientific basis for pharmacokinetics and pharmacodynamics of drugs is evident for this group of people, thus facilitating the emergence of serious and unpredictable adverse reactions,^8^ especially in view of the vulnerability in which NBs are in NICU.

Studies in several countries,^9^ ^,^ ^10^ ^,^ ^11^ ^,^ ^12^ ^,^ ^13^ ^,^ ^14^ ^,^ ^15^ including Brazil,^16^ ^,^ ^17^ ^,^ ^18^ have shown the frequent use of OL therapy in NICUs. Although there are regulatory agencies for drug registration, such as the Food and Drug Control Agency (FDA), in the United States, the European Medicines Agency (EMA), for European countries and, in Brazil, the Brazilian Health Regulatory Agency (ANVISA), there is still no specific regulation for the neonatal population, which hinders the attempt to decrease the use of OL medications in these individuals. Lack of regulation for medications in the pediatric population, the dissimilarity in scientific studies on the action of drugs in pediatrics, and lack of knowledge by some professionals of the subject can promote the use, with increasing frequency, of OL medications in this phase of life, which is especially common in the neonatal period. This is largely due to the ethical difficulty in research and the biological impact that drugs can bring to this vulnerable population.

Therefore, a paradox between encouraging scientific research involving newborns, which aim to learn about the efficacy and safety of drugs, and preserving ethical issues related to their intrinsic vulnerability exists. In view of this, present research aimed to evaluate the frequency of OL medications prescribed for newborns admitted to the ICU, to compare data to those of an international agency and verify the association of the number of these medications with the clinical variables of these patients. With that, we can bring more recent and objective knowledge to a topic of such relevance, helping to reduce damage to this population, which is already subject to the risks inherent to the severity of the pathologies that led to hospitalization and to the countless necessary procedures during their treatment.

## METHOD

A cross-sectional study was carried out, with data collection directly from the medical records of neonates admitted to the NICU of a reference maternity in high-risk pregnancies, located in Aracaju City, Sergipe State. It was a non-probabilistic, convenience sample, with records selected consecutively from June 2017 to April 2018. NBs aged 0 to 28 days were included, with a minimum hospital stay of one day, who needed to use invasive mechanical ventilation. Those from other institutions, NBs with second admission in the study period, and those who did not contain all the necessary data in the medical record were excluded.

Data were collected as from the first day of hospitalization to seven days after the suspension of mechanical ventilation. For their organization, an online form was generated, which was filled out according to the medical records of each newborn admitted to the NICU during the study period. No intervention was made by the data collection team in relation to patients, so that prescription or medical record patterns were not modified.

The variables studied were: anthropometric measurements at birth, their classification by INTERGROWTH-21^st^, Apgar score, epidemiological variables (sex, gestational age, and type of delivery), reason for hospitalization, complications during hospitalization (anemia, apnea, atelectasis, need for reintubation, pulmonary hypertension, infection, sepsis, pneumothorax, pneumonia, respiratory failure, drop in saturation, and death), and medications administered.

The Anatomical Therapeutic Chemical (ATC) classification,^19^ adopted by the WHO, was used to categorize medications at different levels, according to the organ or system on which they act and to their chemical, pharmacological, and therapeutic properties. Medications are then separated into five levels: 1 - anatomical, 2 - therapeutic, 3 - pharmacological, 4 - chemical, and 5 - chemical substance. In the present paper, classification at the first level was used, which is subdivided into: A - alimentary tract and metabolism; B - blood and blood-forming organs; C - cardiovascular system; D - dermatological; G - genitourinary system and sex hormones; H - systemic hormonal medications, except for sex hormones and insulins; J - antiinfectives for systemic use, L - antineoplastic and immunomodulatory; M - musculoskeletal system; N - nervous system; P - antiparasitic products, insecticides, and repellents; R - respiratory system; S - sensory organs; and V - various.

The ANVISA^20^ electronic drug description leaflet was used to assess medications. Those whose prescription was not in accordance with the marketing authorization issued for their age and those that did not have drug description leaflets were considered OL. The classification of drugs according to FDA criteria was carried out using the electronic drug description leaflet^21^ to compare our results to those of an international institution.

Variables were described with the median and interquartile range (IQR), whenever they were continuous or discrete, and with absolute and relative percentage frequency, whenever they were categorical. Associations between categorical variables were tested using Pearson’s chi-squared test. Differences in position measurement were tested using the Mann-Whitney U test and Poisson test. Poisson regression was applied to assess which factors influenced the greatest number of drugs. Thus, variables with p<0.2 in the Poisson test were included in the model and selected with the backward selection method. The software used was the R Core Team 2018, and the level of significance adopted was 5%.

Research was approved by the Research Ethics Committee of Universidade Tiradentes, under Presentation Certificate for Ethical Appreciation (Certificado de Apresentação para Apreciação Ética - CAAE) No. 68001817.5.0000.5371.

## RESULTS

The sample consisted of 158 newborns. Of these, 95 (60.1%) were male, and slightly more than half (58.2%) were born via C-section. The average gestational age was 32.5 weeks, and the birth weight, 1,774 g. Sample characterization is presented in Table 1. The most common diagnoses at admission were respiratory distress (80.3%), jaundice (72.7%), infection (49.3%), sepsis (23.4%), and pneumonia (15.1%).

**Table 1:** Clinical-demographic data of patients admitted to the Neonatal Intensive Care Unit, from June 2017 to April 2018.

	n (158)	%
Sex		
Male	95	58.3
Classification of gestational age at birth		
Full term	19	12.2
Late preterm (34 to 36 weeks)	20	12.9
Early preterm (<34 weeks)	119	74.8
Mean (min-max) birth weight (kg)	1.7 (0.6-4.9)	
Classification of birth weight		
Adequate	9	5.9
Insufficient	12	7.5
Macrosomic	5	3.1
Low weight	55	34.8
Very low weight	56	35.4
Extreme low weight	21	13.2
Apgar at 5^th^ minute		
No asphyxia	129	81.6
Mild asphyxia	24	15.1
Moderate asphyxia	4	2.5
Type of delivery		
Natural	66	41.7
C-section	92	58.2

A total of 61 types of medications was used during the study period, and 1,167 prescriptions were recorded. All the newborns examined used some medication, with an average of 7.3 per neonate. The most prescribed therapeutic class was antiinfectives for systemic use (class J), followed by medications for the nervous system (class N), respiratory system (class R), and cardiovascular (class C). Among these classes, those with the highest number of OL medications were, in decreasing order: class J (antiinfectives), class N (nervous system), and class A (alimentary tract and metabolism). Dipyrone, acetaminophen, and succinylcholine were categorized as unclassified drugs because they are not included in the ATC classification. The number of drugs per class is described in Table 2.

**Table 2:** Number of most used medications by class and by patients in the Neonatal Intensive Care Unit, June 2017 to April 2018.

	Medications/patient		No. of medications	No. of patients	% of patients	Off-label	
	Average	SD				n	%
Medications used	7.3	3.6	1167	158	100		
Off-label medications	2.7	1.8	440	155	98.1	155	100
Class A	0.4	0.6	64	49	31.0	25	26.1
Class C	0.6	0.9	109	71	44.9	12	7.7
Class H	0.2	0.4	45	45	28.5	0	0.0
Class J	3.7	2.0	590	155	98.1	150	96.8
Class N	1.3	1.1	205	119	75.3	76	49.0
Class R	0.5	0.7	91	80	50.6	4	2.6
No classification	0.1	0.3	20	18	11.4	18	11.6

SD: standard deviation; A: medications for alimentary tract and metabolism; C: medications for the cardiovascular system; H: systemic hormonal medications, except for sex hormones and insulins; J: antiinfectives medications; N: medications for the nervous system; R: medications for the respiratory system.

Of the 1,167 prescriptions, 440 (37.7%) were made OL, according to ANVISA, and 484 (41.5%) according to the FDA. Almost all NBs (98.1%) were exposed to at least one OL medication by ANVISA, totaling an average of 2.7±1.8 per NB. Regarding the FDA, 78.5% of newborns were exposed, and the average was 3.0±2.7 OL medications per patient. According to ANVISA, the most used OL medication were: ampicillin (91.7%), fentanyl (37.9%), metronidazole (20.2%), and cefepime (16.4%); according to the FDA, they were amikacin (39.2%), oxacillin (38.6%), fentanyl (37.9%), and vancomycin (27.2%).

Of the 61 types of medications found, six (9.8%) contained, information for adult use only, according to the drug prescription leaflet by ANVISA. Information on pediatric use (not including neonates) was found in 26 (42.6%) drug prescription leaflets. In 28 (45.9%), guidelines for use in neonates were observed, and 11 (18%) specified use in premature infants. There was also a medication that did not have a drug prescription leaflet (multivitamin). Information from the FDA’s electronic drug prescription leaflet showed that six (9.8%) drugs contained information for adult use only, 18 (29.5%) reported adult and pediatric use (not including neonates), 29 (47.5%) contained information for use in neonates, with their use in preterm infants specified in 18 of them (29.5%). In addition, eight (13.1%) were not found in the FDA’s electronic drug prescription leaflet.

There was no statistically significant association between the presence of complications during hospitalization and the number of OL medications (2 *versus* 2; p=0.574), with the exception of respiratory failure (6 *versus* 2; p=0.001), and pneumonia (2 *versus* 2; p=0.024), which, separately, were associated to the number of these medications, as shown in Table 3. There was a reduced number of deaths (5%), whose main cause was septic shock. No association between the number of deaths and the number of OL medications (2 *versus* 2; p=0.250) was found.

**Table 3:** Complications during hospitalization and use of off-label medications of patients admitted to the Neonatal Intensive Care Unit, from June 2017 to April 2018.

	No. of off-label medications			***p*-value W**	Poisson test
	Median	IQR	n		
Complications					
Yes	2	1.3-4	112	0.574	0.592
No	2	1-4	46		
Reintubation					
Yes	2	1-4	56	0.990	0.996
No	2	1-4	102		
Anemia					
Yes	2	1.5-2.5	13	0.401	0.283
No	2	1-4	145		
Apnea					
Yes	2	1-4	28	0.583	0.535
No	2	1-4	130		
Atelectasis					
Yes	3	2-4.5	14	0.089	0.066
No	2	1-4	144		
Shock					
Yes	4	2-0	3	0.201	0.205
No	2	1-4	155		
Respiratory stress					
Yes	2	1-5	31	0.524	0.659
No	2	2-4	127		
Edema of the glottis					
Yes	3	1.8-4.3	10	0.431	0.417
No	2	1-4	148		
Infection					
Yes	2	1-3	26	0.158	0.065
No	2	1-4	132		
Respiratory failure					
Yes	6	1.5-7.5	5	0.113	0.007
No	2	1-4	153		
Pneumonia					
Yes	2	1-2	9	0.024	0.025
No	2	1-4	149		
Sepsis					
Yes	2	1.5-2.5	9	0.227	0.100
No	2	1-4	149		
Death					
Yes	2	1-2.8	8	0.250	0.253
No	2	1-4	150		

IQR: interquartile range; n: absolute frequency; W: Mann-Whitney test.

Multivariate association showed that the use of oxygen for five to seven days or for more than 12 days, with complications such as atelectasis and respiratory failure, was associated to a greater number of OL medications in late preterm infants (Table 4).

**Table 4:** Poisson regression model for off-label medications offered to patients admitted to the Neonatal Intensive Care Unit, June 2017 to April 2018.

	Exp. (B_adj_)	95%CI	***p*-value**
Gestational age			
Early preterm	1		
Late preterm	1.51	1.15-1.99	0.003
Full term	1.30	0.97-1.74	0.074
Days on O_2_			
Did not use	1		
1 to 4 days	0.85	0.65-1.11	0.244
5 to 7 days	2.36	1.58-3.54	<0.001
8 to 12 days	1.31	0.58-2.96	0.513
>12 days	3.06	1.44-6.51	0.004
Atelectasis			
Yes	1.63	1.20-2.21	0.002
No	1		
Respiratory failure			
Yes	1.98	1.30-3.01	0.001
No	1		

Exp.: exponential; B_adj_: estimate of the adjusted parameter of Poisson regression; 95%CI: 95% confidence interval.

## DISCUSSION

The results of the present study show that a large part of the sample was exposed to OL medications, both by ANVISA and the FDA, (98.1% *versus* 78.5%), corroborating a series of research throughout the world that reveal figures as large as those evidenced in our prescriptions. A recent Brazilian prospective study analyzed 17,421 prescription items in 220 NBs during a year in a NICU in Natal City, Rio Grande do Norte State, based on FDA determinations. It concluded that 49.3% of the prescriptions were OL, and 96.4% of hospitalized NBs received these drugs.^18^ A smaller Spanish retrospective study, carried out in a NICU at a university hospital, found that 41.4% of prescriptions were for OL medications, and 90.2% of NBs received at least one of them.^12^ Another Spanish study compared the use of OL medications in children older than 28 days to their use in newborns and concluded that the number of OL prescriptions was higher in the group of neonates (8.3 *versus* 6.1 drugs), which confirms the high frequency of these drugs early in life.^22^ Indian research involving 460 newborns showed that, of the 2,642 prescription items, only 326 (12.3%) were used OL.^14^ This prospective analysis is the one that most distance from our results, with the OL status determined by an independent British agency regulator and considered when there was a deviation in relation to the indications, doses, dosage forms, frequency of administration, and age. On the other hand, a six-month retrospective cohort study, carried out at the NICU in Brazil with 192 newborns, obtained a total of 3,290 prescriptions, of which 3,145 (95.6%) were made OL.^16^

Although there is consensus among studies as to the high frequency of using OL medications in the NICU, the differences between results may be related to the different definitions of the term OL and the choice of the regulatory agency used. In the present study, the recent definition of OL medication, according to Aronson and Ferner,^3^ and the regulation for drugs in force in Brazil, by ANVISA, was adopted. In our study, less than half of the medications contained information in drug description leaflets for use in newborns, and even less for premature infants, using both the ANVISA and the FDA guidelines to assess that. In addition, although some drug description leaflets show that the medication is for pediatric use, they do not present information about its application in the first 28 days of life, generating conflict of information: containing the pediatric name, but not addressing neonatology. Drug description leaflets normally generalize the term pediatric, without determining age and or including specifications for neonates, premature and low birth weight, increasing the number of OL prescriptions.

Given the need for use, the experience of the interventionist pediatrician, or the service protocol, the authorization by the regulatory agency for the use of the medication is overridden, because most drugs are not officially indicated for use in newborns. A Chinese study in Shanghai, with 679 questionnaires answered by pediatricians, pharmacists, nurses, and administrators with pediatric qualifications in 69 hospitals, showed that almost half of pediatricians acknowledged having prescribed OL medications and concluded that the main reason for these prescriptions was the lack of information of pediatric dosage in the drug description leaflet,^15^ contributing to the fact that this absence was also the most common OL prescription status. An Israeli study pointed out that, OL prescriptions in the NICU have not decreased in recent decades; maybe, they have even increased. This seems to occur because most drugs used in newborns are old and have no patent, which limits incentives for measures that regulate these products. Furthermore, when there is regulation, there is a delay between the beginning of the effectiveness of regulatory measures and the entry or withdrawal of products on the market.^23^ In the present study, most OL medications have also been used for a long time and do not have a patent, contributing to their pediatric regulation difficulties.

Just like the article herein presented, several studies have shown that the newborns who suffer most from these prescriptions are premature and of low birth weight,^11^ ^,^ ^12^ ^,^ ^13^ ^,^ ^14^ ^,^ ^16^ ^,^ ^17^ ^,^ ^18^ because this population is the majority in the NICU.^24^ ^,^ ^25^ Given that at the beginning of life glomerular filtration is lower in relation to adulthood this is an important fact. In addition, drug’s clearance is variable, depending on the genetic (mainly ontogeny), environmental and constitutional characteristics of the newborn.^26^ Besides that, premature and underweight newborns have less muscle and adipose tissue, a higher concentration of water and less protein, with less binding and transport capacity between them.^27^ In these cases, the concern is not only with unauthorized use, but with the number of medications consumed by the NB, as there may be drug interactions and increased side effects in their immature organism.

In our study, the group of drugs most used in the NICU was that of antiinfectives. Gentamicin, widely used in the neonatal period, was considered the OL medication most used in similar studies.^18^ ^,^ ^28^ However, it is a drug authorized for use in newborns by ANVISA. Thus, in the present study, ampicillin was the most prescribed OL antibiotic. This medication is part of the first-line drug (gentamicin and ampicillin) in the treatment of early neonatal sepsis,^29^ which reinforces the need for studies on the adverse effects and safety of this drug in neonatology.

The group of neurological drugs ranks second as to the most prescribed drugs in our study. Fentanyl, the most used opioid analgesic in neonatology, was the most frequent. Such observation is probably because sample is composed of NBs who underwent the intubation procedure. High doses of this drug lead to muscle stiffness and, when they affect the breathing muscles, they can affect ventilation and even lead the patient to death.^30^ Nonetheless, Lee et al. prospectively analyzed adverse effects of 32 drugs over a year in NBs admitted to the NICU and did not show alarming numbers of fentanyl side effects.^10^

In the present study, although no association was found between the number of OL medications prescribed and the presence of complications during hospitalization, in isolation, a greater number of these drugs were used in premature infants who had complications such as atelectasis and respiratory failure. The appearance of these conditions, in this group of patients, can be due to the fact that the main causes of hospitalization in the NICU are, precisely, prematurity and diseases of the respiratory system.^18^ In addition, 100 % of medications assigned to the respiratory system were used in an OL way in our study.

A retrospective Korean study analyzed 5,130 prescriptions in 2,779 pediatric patients, including neonates, and demonstrated that children who died received a greater number of OL medications. However, there was no direct association between the two variables, which suggests that the severity of the disease is what would be related to the number of medications used, including those used OL, and also to the cause of death.^10^ Moreover, the present investigation did not show an association between increased frequency of deaths and the number of OL medications administered. Deaths and their causes have not been addressed in other studies like this, which is a differential of the present study.

Even with the study limitation, which was that research was carried out in a single health unit, our study results correspond to the reality found in other studies. The scenario shown suggests that ANVISA must carry out an inspection of drug description leaflets to offer more support to professionals who make the prescriptions and, above all, more safety for patients who need to use medications. In view of the ethical difficulties in conducting clinical trials for this purpose, this type of retrospective study on OL medications in neonatology could be used by regulatory agencies for the review of drug description leaflets.
